# Supplementary material for: General scaling in bidirectional flows of self-avoiding agents
Source: Sci Rep. 2019 Dec 6;9:18488. doi: 10.1038/s41598-019-54977-3 (PMC6898712; doi:10.1038/s41598-019-54977-3)
Supplement: Supplementary file 1 — Supplementary Information [file 41598_2019_54977_MOESM1_ESM.pdf]

General scaling in bidirectional flows of self-avoiding agents
Supplementary Information

Javier Cristín, Vicenç Méndez and Daniel Campos
Grup de Física Estadística. Dept. de Fisica. Universitat Autonoma de Barcelona
08193 Bellaterra (Barcelona) Spain
(Dated: October 1, 2019)

In this Supplemental Material we display further evidence for the phase transition and the universal interaction explained in the main text.

DISTRIBUTION FUNCTION

For the sake of completeness, we show at the Fig. 1 the equivalent to the partial distribution analysis (main text, Fig. 4) but now for the lanes state. The splitting in different ranges of v_r in this case has to be necessarily different to that for the disordered state due to the lack of intermediate relative velocities when the lanes are formed. All agents will be moving either leftwards or rightwards (according to the preferred direction of each). Then relative velocities are either very low ($v_r < 1.5$, for agents in the same lane) or very large ($v_r > 1.5$, for agents in lanes with opposite directions), while there is no statistics at the intermediate regime $1 < v_r < 2$ used in Figure 4 in the main text. Additionally, we often observe that particles persistently moving in the same lane and very close to each other (so, with $\tau \to 0$) artificially dominate the statistics, so these have been removed when computing $g^{\dagger}(\tau)$ in Fig. 1.

The collapse observed in the distribution $g^{\dagger}(\tau)$ for different v_r ranges confirms the behavior already reported for the disordered phase (note that now even the repulsive case satisfies the collapse to a great extent). This further supports the existence of an underlying phenomena enhancing the prominence of the τ-space whenever self-avoidance and bidirectionality effects drive the system dynamics.

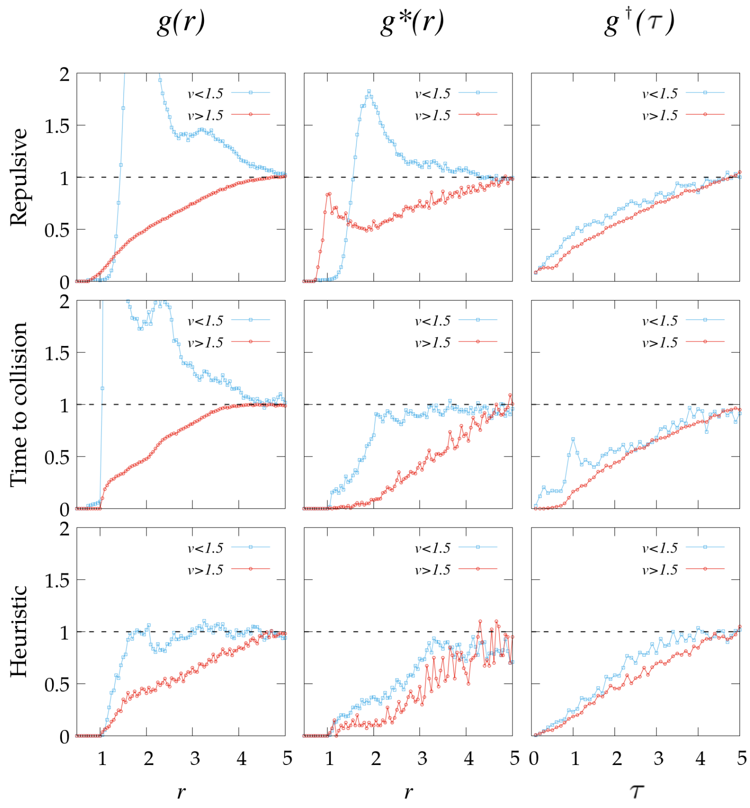

FIG. 1. *Radial and partial distribution functions $g(r)$, $g^*(r)$ and $g^{\dagger}(\tau)$ when split into different regimes according to the relative speed between pairs v_r. Results are shown for $\rho = 0.14$ and $\xi = 4$, which corresponds to a lane state. The repulsive interaction is fixed to $k = 4$*

I. BEHAVIOUR OF THE REPULSIVE POTENTIAL

While in the Figure 4 of the main text we present the results for the repulsive potential with $k = 4$ for the sake of simplicity, we here complete the comparison study by showing that other values of k lead to very similar results (in particular the cases $k = 2$ and $k = 3$ are shown). We compare the distribution functions $g(r)$, $g^*(r)$ and $g^{\dagger}(\tau)$

(Fig. 2) when they are split according to the different values of the relative speed v_r (Fig. 2). In agreement with our discussion in the main text, we find that the curves tend to converge in the case where the τ-space is considered. Although the convergence is far from being perfect (as is to be expected, actually, since $F_{rep}^{(sa)}$ is not defined in the τ-space), we still find that the effective interaction potential, defined through $V(\tau) \sim \ln\left[g^{\dagger}(\tau)\right]$, still satisfies the same scaling $V(\tau) \sim \tau^{\gamma}$ with $\gamma \approx 2$ in the disordered state and $\gamma \approx 1$ for lanes. As a whole, this analysis confirm the universal nature of the scaling reported for $V(\tau)$, which seems to be independent of the self-avoiding mechanism considered, even for those cases where this mechanism has nothing to do originally with the τ-space.

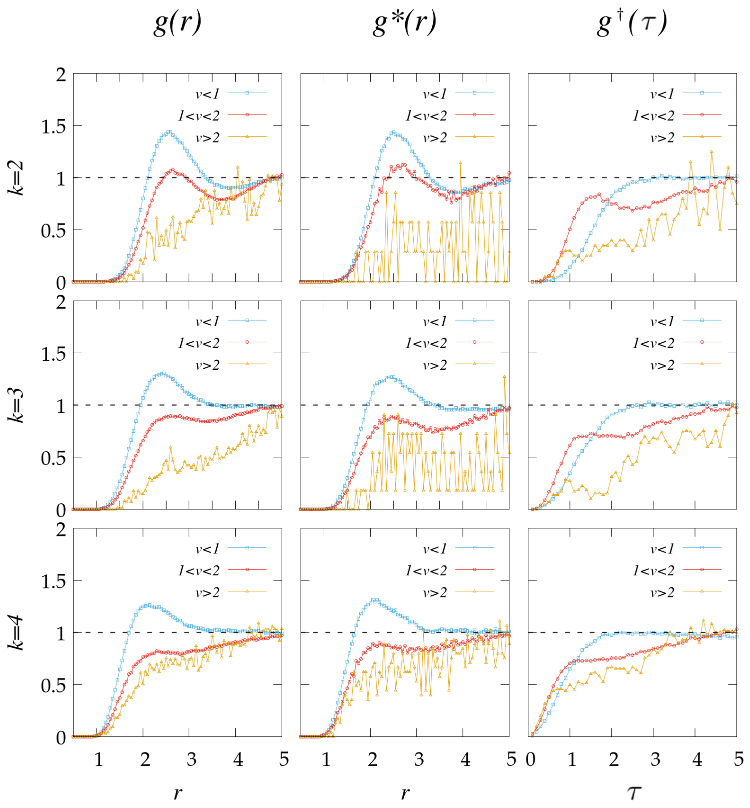

FIG. 2. *Distribution functions* $g(r)$*,* $g^{*}(r)$ *and* $g^{\dagger}(\tau)$ *when split into different regimes according to the relative speed between pairs* v_r*. The example system is formed by agents interacting with the repulsive potential with a)* $k = 2$ *b)* $k = 3$ *c)* $k = 4$ *at the phase space point* $\rho = 0.14$ *and* $\xi = 0.1$*.*

In the Figure 4 of the main text we have already proved that $g(r)$ exhibits very different shapes when data is split according to the relative speed between the pairs of particles interacting. However, at least for the case of the repulsive potential, where the force between pairs $\mathbf{F}^{sa}$ (and so the corresponding potential resulting from the integration of $\mathbf{F}$) depend only on the distance r, we must expect that $g(r)$ is well-defined (since in this case the classical reversible work theorem holds). Then a real (physical) potential $V_p(r)$ can be properly defined (but this does not necessarily have any formal relation with the effective potential $V(\tau)$ analyzed in the main work). To verify that our system is well-behaved then we include here the behavior obtained for the repulsive case F_{rep}^{sa} when $g(r)$ is split according to the actual value of the particles speed v (and not v_r, as in Figure 4 of the main text). The overlap between all the curves (independent of v) confirms that our simulated system is physically sound, and so supports the idea that the general behavior reported for $V(\tau)$ is not an artifact.

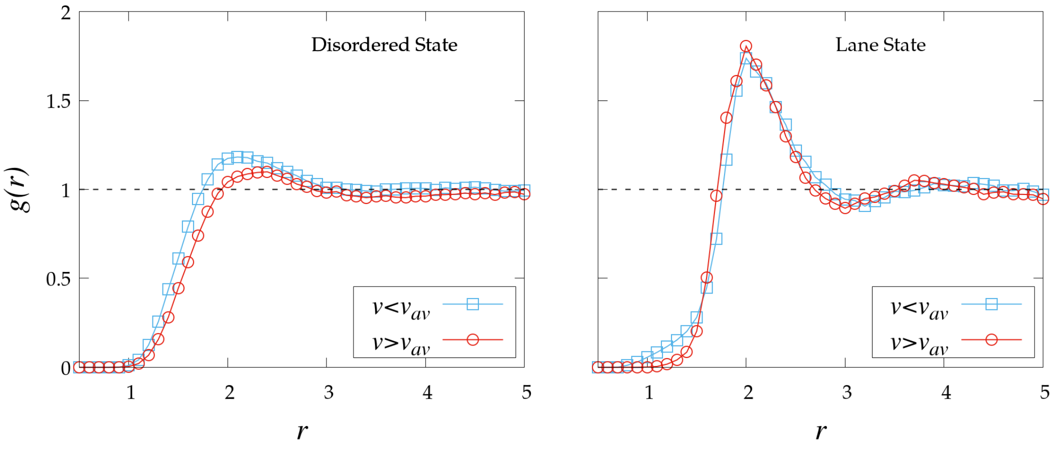

FIG. 3. *Radial distribution function $g(r)$ when split into two different regimes according to the particle speed v. The left image corresponds to $\xi = 0.1$ and the right image for $\xi = 2$, with $\rho = 0.14$ and $k = 4$ in both cases.*
